# Consumption of energy drinks among adolescents in Norway: a cross-sectional study

**DOI:** 10.1186/s12889-018-6236-5

**Published:** 2018-12-19

**Authors:** Naim Degirmenci, Ingrid Nesdal Fossum, Tor Arne Strand, Arild Vaktskjold, Mads Nikolaj Holten-Andersen

**Affiliations:** 1 Department of Pediatrics, Innlandet Hospital Trust, Anders Sandvigsgate 17, 2609 Lillehammer, Norway; 2 Department of Research, Innlandet Hospital Trust, Lillehammer, Norway; 3 Centre for International Health, University of Bergen, Bergen, Norway; 4 Institute of Public Health, Inland Norway University of Applied Sciences, Elverum, Norway; 5 Department of Clinical Medicine, University of Oslo, Oslo, Norway

**Keywords:** Epidemiology, Adolescents, Lifestyle, Energy drinks, Caffeine, Survey, Norway

## Abstract

**Background:**

Energy drink (ED) consumption is increasing all over the world. We sought to describe the consumption of EDs among adolescents in Norway, and to explore the determinants of daily and high consumption.

**Methods:**

Population-based cross-sectional data were collected from a sample of 31,091 secondary school students in grade 8–13 aged 12–19 years. School grade, residency, socioeconomic status (SES), physical activity and leisure screen time were included in multiple regression analyses, in order to investigate their associations with daily and high (≥four times weekly) ED consumption.

**Results:**

52.3% of the respondents were ED consumers and 3.5% were high consumers. Boys consumed twice as much ED as girls (boys: 36.3 ml/day, girls: 18.5 ml/day, geometric means), and the proportion of male high consumers was 3.7-times higher than that of females. The adjusted odd ratio (OR) of upper secondary school (grades 11–13, ages 15–19) students being high ED consumers were higher than for lower secondary school (grades 8–10, ages 12–15) students (OR 1.1(confidence interval (CI):1.0–1.3)), as well as higher for rural than urban residents (OR 1.3 (CI: 1.1–1.5)). Gradients for the increased ORs of being a high ED consumer were found for decreased SES, decreased frequency of physical activity and increased daily leisure screen time.

**Conclusions:**

More than half of the respondents reported that they were ED consumers. Daily and high consumption were independently associated with male gender, physical inactivity, high leisure screen time, low socioeconomic status and rural residency.

**Electronic supplementary material:**

The online version of this article (10.1186/s12889-018-6236-5) contains supplementary material, which is available to authorized users.

## Background

Over the past few years, there has been a rapid increase in the consumption of energy drinks (ED) globally, with children and adolescents being the major consumers [1–3]. EDs are marketed as boosters of mental and physical capacity, and their marketing is often directed at young people [4]. Worldwide attention has been focused on the potentially negative side effects of EDs on consumers’ health, since EDs contain high levels of caffeine and sugar in combination with minerals, vitamins, amino acids and herbal supplements. Numerous reports of the adverse effects of ED intake have described a variety of symptoms and affected organ systems, including tachycardia, hypertension, confusion, agitation, seizures, liver damage, kidney failure and cardiac dysfunction, with potential deadly outcomes [2, 5]. Recent studies have demonstrated significant haemodynamic changes in healthy young individuals following ED consumption, with elevated systolic and diastolic blood pressures, increased cardiac output and myocardial load, repolarization abnormalities and reduced cerebral blood flow velocity [6–8], as well as a significant increase in circulating catecholamines, reflecting sympathetic activation [8].

Attention has specifically been directed towards ED consumption among children and adolescents. Studies have shown that among these groups, ED consumption has been associated with health complaints and sleep problems [1, 9–13], as well as with problems with behaviour regulation, metacognition, school performance [14–16], executive functions [12] and the risk of being overweight or obese [17]. It has also been reported that ED consumption among adolescents is correlated with various adverse behavioural patterns, including alcohol and substance abuse and risk behaviours [12, 18]. Furthermore, ED consumption has been associated with emotional difficulties, lower subjective well-being and symptoms of depression and anxiety [12, 18]; it has even been associated with increased odds of suicide attempts among 13–18 year olds, adjusted for possible confounding factors including stress, sleep and school performance [15].

Details on the causative mechanisms of the variety of adverse effects of EDs remain elusive [2]. In 2013, a French official expert opinion statement concluded that a substantial proportion of the adverse effects reported among French ED consumers were likely to be or probably were caused by EDs [19]. The extent to which the outcomes of ED use are due to caffeine, to other ED constituents or to an interaction between the two is currently unclear; however, research suggests that the harmful outcomes that can be caused by EDs exceed the direct effects of caffeine alone [20]. Adverse events have occurred following the consumption of highly variable volumes of ED, suggesting that some individuals are more susceptible to the effects of EDs than others [19]. Children and adolescents may be at increased risk of the negative effects of ED consumption as a result of their low body mass and higher caffeine sensitivity, thus resulting in their greater vulnerability to the effects of caffeine and, therefore, EDs [21]. Studies have shown that EDs are used by large proportions of adolescents, and that children down to 10 years of age also consume ED [1, 9, 10, 18]. Male gender, rural residency, lower educational background and time spent in front of a screen have in individual studies been shown to be associated with ED consumption [1, 9, 10, 22–25].

The aim of the study was to survey the extent of ED consumption among adolescents in Norway, as well as to assess the impact of sociodemographic and lifestyle factors that may be associated with the risk of high consumption.

## Methods

### Study design and participants

Data for the study were collected through the Ungdata survey, which is a national, anonymous data collection survey carried out among adolescents in Norway on a municipal level. Its aim is to survey adolescent health and well-being (for more information on the Ungdata survey, see ungdata.no). Participation is on a volunteer basis and is free of charge, and the adolescents or their parents are free to opt out. The survey is conducted annually and municipalities are encouraged to participate every 3 years, ensuring that all adolescents get to participate once during their lower and once during their upper secondary schooling. The participating municipalities decide which grades they want to include. The questionnaire is filled out by the participants online during school hours with teachers present in the class. The study was approved by the Norwegian Centre for Research Data.

**Fig. 1:**
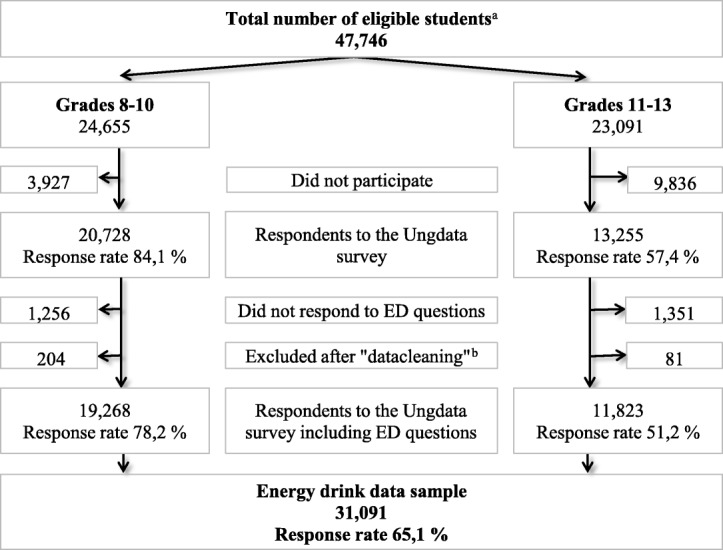
Flow chart and response rates in a study on ED consumption among secondary school students^a^Eligible students from municipalities participating in the Ungdata survey in 2015-2016 where the ED questionnaire was included. ^b^Due to lack of answers or unlikely combinations of answers on given defined indicators [35], 285 out of 31,091 respondents were excluded from the analyses

The data used in the present study were collected in 2015 and 2016, from adolescents in the lower and upper secondary education system (grades 8–10 including ages 12–15 years and grades 11–13 including ages 15–19 years, respectively). Thirty-one thousand and ninety-one adolescents between the ages of 12 and 19 years (46.9% male, 50.0% female, 3.1% missing) responded to the questionnaire, including the elective module containing questions on EDs. Statistics from the 63 municipalities (2015: 41 municipalities; 2016: 22 municipalities) that chose to participate in 2015 and 2016 showed that 47,746 adolescents were eligible to participate in the survey, with an approximately even distribution between lower and upper secondary schools (Fig. 1). The average response rates were 78.2% in lower secondary schools and 51.2% in upper secondary schools (Fig. 1). In Norway, children start school the year they turn 6 years old and attending school is obligatory by law until the completion of grade 10 (normally at the age of 15 or 16). Further education is voluntary, however statistics show that the majority of 16–18 year-olds (92.3% in 2017) go on to attend upper secondary schools [26].

### Measures

The Ungdata electronic questionnaire used in this study included 272 questions [27]. In 2015 and 2016 the survey contained a fixed section including 167 questions, as well as elective modules including 105 questions, of which 9 concerned ED consumption. A set of variables from the Ungdata survey were selected a priori for the analyses in the present study. These were ED consumption, grade, gender, residency, SES, frequency of physical activity and leisure screen time.

ED consumption was assessed using two items: “How often do you drink energy drinks?” with options on a seven-point scale from “never” to “daily”, and “How much energy drink do you usually drink when you do consume them?” with options on a six-point scale from “one small can (ca 250 ml)” to “several cans corresponding to more than 1.5 litres”, both of which had mutually exclusive increments. This enabled the calculation of adolescents’ average daily ED intake. Respondents were defined as ED consumers if they reported consuming EDs from daily to once a month or less.

Leisure screen time was assessed by the question “Outside school, how much time do you normally spend on activities that involve looking at a screen (TV, computer, tablet, mobile phone) each day?”, with options on a seven-point scale ranging from “none” to “more than six hours” with mutually exclusive increments. The frequency of physical activity was assessed by the question “How often do you engage in activities making you sweat or feeling out of breath?”, with options on a six-point scale ranging from “never” to “at least five times a week”, with mutually exclusive increments. Based on official statistics from 2015, Norway’s municipalities were defined as urban or rural according to a number of inhabitants equal to or above 20,000 (urban residency) or below 20,000 (rural residency).

### Statistical analyses

Regarding ED consumption, we first calculated adolescents’ average daily ED intake (ml/day) by multiplying the self-reported frequency (number of days per 4 weeks) with the self-reported volume of EDs consumed (ml), averaged over 28 days. Next, categories of ED consumer were defined accordingly: infrequent consumers (less than once weekly), frequent consumers (one to three times weekly) and high consumers (four times weekly or more) [9]. Finally, daily ED consumption was log_e_ transformed to attain normal data distribution.

Regarding leisure screen time, three options (“none”, “less than one hour” and “one to two hours”) were all merged into the one response of “less than two hours” due to the relatively low number of respondents falling into the lower categories. Similarly, in terms of frequency of physical activity, two options (“seldom” and “once to twice a month”) were merged into the response “seldom”, while three options (“once to twice per week”, “three to four times per week” and “at least five times per week”) were merged into the response “often”, thereby changing the scale from a six-point to a three-point one. We found no differences in frequency or volume of ED consumption between the three physical activity subgroups that were merged into the group “often”. Especially, we did not find increased ED consumption in the group with highest level of activity (data not shown). SES was assessed on a five-point scale, based on a compound score encompassing three dimensions: educational level of parents, number of books at home and family affluence scale II (car, bedroom, holiday and computer status of the household) [28, 29]. Details are described in Additional file 1.

IBM SPSS version 23 was used to calculate the geometric mean, 95% confidence intervals (CI), the median and the interquartile range. We used multiple linear regression in STATA version 15 (College Station, Texas) to compare ED intake between the different study categories. The back-transformed regression coefficients from the linear models are geometric mean differences, and represent the ratios between the exposure variable categories. We used multiple logistic regression models to estimate the associations between the selected exposure variables and being a high ED consumer. The following independent variables were included in the regression analyses: grade, gender, residency, SES, frequency of physical activity and leisure screen time. These analyses were also undertaken by stratifying on grade and gender.

## Results

There was an approximately even distribution between boys and girls, as well as between urban and rural residency. The main baseline features are listed in Table 1.

**Table 1:** Descriptive sample characteristics in a study on ED consumption in Norwegian adolescents.

Variable	n (%)
School level	
Lower secondary school^a^	19,268 (62.0)
Upper secondary school^b^	11,823 (38.0)
Grade	
8	6316 (20.3)
9	6117 (19.7)
10	6469 (20.8)
11	4934 (15.9)
12	4118 (13.2)
13	2705 (8.7)
Missing	432 (1.4)
Gender	
Female	15,530 (50.0)
Male	14,581 (46.9)
Missing	980 (3.1)
Residency^c^	
Urban	14,394 (46.3)
Rural	16,697 (53.7)
Frequency of physical activity^d^	
Often	25,791 (83.0)
Seldom	4351 (14.0)
Never	573 (1.8)
Missing	376 (1.2)
Leisure screen time	
Less than two hours	6755 (21.7)
Two-three hours	7117 (22.9)
Three-four hours	7614 (24.5)
Four-six hours	5427 (17.5)
More than six hours	3840 (12.4)
Missing	338 (1.1)
Basis for calculating socioeconomic status index:	
See Additional file 1	

*n* = 31,091

^a^Lower secondary school includes grades 8–10 and ages 12–15 years ^b^ Upper secondary school includes grades 11–13 and ages 15–19 years ^c^ Urban residency: municipalities with ≥ 20,000 residents, rural residency: municipalities with < 20,000 residents^. d^ Often: once a week or more, seldom: once to twice a month or less

### ED consumption

Table 2 shows details concerning the respondents’ self-reported ED consumption. It reveals that 52.3% of them reported that they were ED consumers (39.3% infrequent consumers, 9.5% frequent consumers and 3.5% high consumers). The proportions of boys who were frequent or high ED consumers were more than three times higher than the corresponding proportions of girls.

**Table 2:** Distribution of ED consumer categories and weekly number of cans consumed for girls and boys

	Total	Girls	Boys	Missing gender
	(*n* = 31,091)	(*n* = 15,530)	(*n* = 14,581)	(*n* = 980)
Consumer categories				
Has never consumed ED	10,869 (35.0)	7341 (47.3)	3170 (21.7)	358 (36.5)
Has previously consumed ED, but has quit	3964 (12.7)	1865 (12.0)	1999 (13.7)	100 (10.2)
ED consumer categories	16,258 (52.3)	6324 (40.7)	9412 (64.5)	522 (53.2)
Infrequent consumers	12,218 (39.3)	5413 (34.9)	6430 (44.1)	375 (38.3)
Frequent consumers	2950 (9.5)	685 (4.4)	2167 (14.9)	98 (10.0)
High consumers	1090 (3.5)	226 (1.5)	815 (5.6)	49 (5.0)
Weekly number of cans^a^ consumed				
No ED consumption	14,833 (47.7)	9206 (59.3)	5169 (35.5)	458 (46.7)
Less than one can	11,691 (37.6)	5244 (33.8)	6081 (41.7)	366 (37.3)
At least one can	4222 (13.6)	936 (6.0)	3139 (21.5)	147 (15.0)
At least two cans	2576 (8.3)	460 (3.0)	2025 (13.9)	91 (9.3)
At least three cans	1289 (4.1)	244 (1.6)	992 (6.8)	53 (5.4)
At least four cans	1101 (3.5)	190 (1.2)	864 (5.9)	47 (4.8)
Missing	345 (1.1)	144 (0.9)	192 (1.3)	9 (0.9)

ED consumer categories were defined as follows: infrequent consumers (EDs consumed < once weekly), frequent consumers (one-three times weekly) and high consumers (≥ four times weekly)

^a^One can = 500 ml

The geometric means for daily ED consumption and its associations with school level, gender, residency, SES, physical activity and leisure screen time are displayed in Table 3. In the adjusted analyses, we found that boys had a mean daily ED consumption which was twice the mean volume consumed by girls (back-transformed regression coefficient of 1.95, corresponding to a mean difference of 95% (CI: 88–103%)). Upper secondary school students had an 8.7% higher ED consumption than lower secondary school students (CI: 5–13%), while respondents living in rural areas had a 13% (CI: 9–17%) higher mean daily ED intake than urban residents. Furthermore, the adjusted analysis demonstrated a gradient of increasing mean differences in daily ED consumption as SES went from higher to lower. The same increasing gradient in consumption was also observed with increased daily leisure screen time, as well as with decreased frequency of physical activity.

**Table 3:** Estimated geometric mean daily consumption and adjusted mean difference for 15,913 ED consumers

Variable		n	Geometric mean daily ED intake (ml/day)	Mean difference (%)	95% CI
School level					
Lower secondary school^a^		9732	26.9	reference group	
Upper secondary school^b^		6181	29.2	8.7%	[4.7–12.8]
Gender					
Female		6180	18.5	reference group	
Male		9220	36.3	95.2%	[88.2–102.5]
Residency^c^					
Urban		7064	26.0	reference group	
Rural		8849	29.2	13.2%	[9.1–17.3]
Socioeconomic status					
Group 5 Highest		2730	24.9	reference group	
Group 4		3213	25.8	−0.1%	[−5.8–5.9]
Group 3		3182	27.3	7.2%	[1.1–13.7]
Group 2		3371	29.1	13.6%	[7.1–20.4]
Group 1 Lowest		3413	31.6	19.0%	[12.3–26.2]
Frequency of physical activity^d^					
Often		13,124	26.5	reference group	
Seldom		2278	32.0	13.1%	[7.3–19.2]
Never		311	53.5	65.8%	[45.4–89.0]
Leisure screen time					
Less than two hours		2549	20.3	reference group	
Two-three hours		3390	22.9	16.7%	[10.0–23.9]
Three-four hours		4058	25.5	29.7%	[22.5–37.3]
Four-six hours		3212	31.0	54.0%	[45.1–63.5]
More than six hours		2561	46.8	116.8%	[103.5–131.0]

*CI* confidence interval

^a^Lower secondary school includes grades 8–10 and ages 12–15 years ^b^ Upper secondary school includes grades 11–13 and ages 15–19 years ^c^ Urban residency: municipalities with ≥ 20,000 residents, rural residency: municipalities with < 20,000 residents. d Often: once a week or more, seldom: once to twice a month or less

### High consumers of EDs

The adjusted odds ratios of being an ED consumer or a high ED consumer, and their associations with grade, gender, residency, SES, physical activity and leisure screen time, are displayed in Table 4. Boys had an adjusted odds ratio of 3.7 (CI: 3.1–4.3) of being a high ED consumer compared to girls, while the adjusted odds ratio was 1.1 (CI: 1.0–1.3) for upper secondary school students compared to lower secondary school students. Participants living in rural areas had an adjusted odds ratio of 1.3 (CI: 1.2–1.5) of being high ED consumers compared to participants living in urban areas. The adjusted analyses demonstrated a gradient in the odds ratio of being a high ED consumer, with increasing odds ratio as the students’ SES decreased from higher to lower. Likewise, gradients could be observed for daily leisure screen time (odds ratio of being a high consumer increasing as screen time increased), as well as for frequency of physical activity (with the odds ratio increasing as frequency decreased).

**Table 4:** ED consumers and high consumers with adjusted^a^ odds ratios (OR)

Variable	Total sample	ED consumers			High consumers		
	n	n	OR	95% CI	n	OR	95% CI
Total sample	31,091	16,258			1090		
School level							
Lower secondary school^b^	19,268	9949	1		638	1	
Upper secondary school^c^	11,823	6309	1.07	[1.02–1.12]	452	1.10	[0.96–1.25]
Gender							
Female	15,530	9412	1		226	1	
Male	14,581	6324	2.62	[2.50–2.75]	815	3.66	[3.14–4.26]
Residency^d^							
Urban	14,394	7218	1		430	1	
Rural	16,697	9040	1.18	[1.13–1.24]	660	1.31	[1.15–1.50]
Socioeconomic status							
Group 5 Highest	6148	2785	1		162	1	
Group 4	6475	3289	1.21	[1.12–1.30]	188	1.03	[0.82–1.30]
Group 3	6145	3236	1.30	[1.20–1.40]	206	1.19	[0.95–1.49]
Group 2	6229	3444	1.44	[1.33–1.55]	244	1.34	[1.08–1.67]
Group 1 Lowest	6084	3499	1.55	[1.43–1.67]	288	1.42	[1.15–1.76]
Frequency of physical activity^e^							
Often	25,791	13,380	1		771	1	
Seldom	4351	2325	0.95	[0.89–1.02]	222	1.42	[1.20–1.68]
Never	573	317	0.93	[0.78–1.12]	62	2.50	[1.84–3.39]
Leisure screen time							
Less than two hours	6755	2619	1		114	1	
Two-three hours	7117	3446	1.53	[1.42–1.64]	145	1.22	[0.94–1.58]
Three-four hours	7614	4138	1.90	[1.77–2.04]	208	1.60	[1.26–2.04]
Four-six hours	5427	3263	2.31	[2.14–2.50]	213	2.18	[1.71–2.76]
More than six hours	3840	2598	3.03	[2.77–3.31]	379	4.92	[3.93–6.16]

*OR* odds ratio, *CI* confidence interval. High consumers were defined as drinking ED four times or more weekly. ^a^ Adjusted for the other variables in the table. ^b^ Lower secondary school includes grades 8–10 and ages 12–15 years ^c^ Upper secondary school includes grades 11–13 and ages 15–19 years ^d^ Urban residency: municipalities with ≥ 20,000 residents, rural residency: municipalities with < 20,000 residents. ^e^ Often: once a week or more, seldom: once to twice a month or less

The regression coefficients were somewhat different when we stratified the analyses by gender and school level (Additional file 2: Table S5, Additional file 3: Table S6, and Additional file 4: Table S7).

## Discussion

In this study, we found that more than half of the participants reported being ED consumers. We also found that male gender, upper secondary school level, low physical activity, increased leisure screen time, rural residency and low SES were determinants for daily ED consumption and for being a high consumer of EDs.

The proportion of adolescents who reported to consume EDs is in line with the results of a large European survey from 2013 (48–82%) [1]. Several large-scale studies on adolescents from other parts of the world have given reports on the proportion of ED consumers; these proportions included, for instance, 35.2% in a study from New Zealand [18], 17.9% in a study from the US [13], 18.2% in a Canadian study [22] and 12% in a Korean study [15]. Overall, there seem to be some differences in rates of consumption in different countries, and the proportions found in our study do not stand out from the rest of the world. It should be noted that any discrepancies in the findings, in addition to reflecting cultural differences, may be a result of methodological differences between the studies [18]. While some of the studies assess the amount of EDs consumed during the last week, others assess ED consumption over the last month or over an even longer period; and for this reason one should be careful when making direct comparisons between the findings reported by single studies. It should also be noted that our study used a rather wide definition of being an ED consumer, as the responses included in this category ranged from consuming EDs daily to once a month or less. This definition corresponds to those applied in other studies [1, 9].

We found higher ED consumption in upper secondary school (grades 8–10, ages 15–19 years) compared to lower secondary school (grades 11–13, ages 12–15 years). Recent studies have shown that children as young as 10 years of age are consuming EDs [9, 10]. It is a cause for concern that the regular consumption of beverages containing high levels of sugar and caffeine, in addition to a variety of other constituents, is being found in younger age groups in many countries. Adolescents have consistently been found to be the age group consuming the highest amounts of EDs [1, 9].

As has been demonstrated in other large-scale cross-sectional studies, we found in our study that male adolescents had a markedly higher level of ED consumption than females; on average, they consumed twice the amount. This finding seems to be largely consistent across studies [1, 9, 10, 13, 22–24]. Independent associations between frequent and high ED consumption on the one hand, and low SES and rural residency on the other, were found in our study. Similarly, findings that reveal associations between ED consumption and rural residency, as well as between ED consumption and the educational background of parents, have been suggested in previous research [9, 24].

Regarding the lifestyle factors examined in this study, we found that there was an inverse association between ED consumption and the frequency of physical activity. In contrast, a study of Saudi adolescents found that those who performed over 60 min of physical activity daily were 1.4 times more likely to consume EDs at least 3 days per week [25]. However, in several studies from the US and Saudi Arabia, no association has been found between ED consumption and level of physical activity [23, 30, 31]. This variation in the results may reflect cultural differences; however, inconsistent findings may also be influenced by methodological differences between the studies, such as the number of participants and differing ways of assessing physical activity.

Furthermore, our results are in line with previous findings concerning an association between ED consumption and time spent in front of a screen [23, 25, 31]. The current findings suggest that adolescents who are heavy screen users have a higher risk of excessive ED consumption. Since screen time has been found to be adversely associated with sleep outcomes [32], as well as related to the symptoms of depression, psychological distress and low self-esteem [33], it is noteworthy that a relatively large proportion (12.4%) of the adolescents in our study reported that they spent at least 6 hours of their daily leisure time in front of a screen.

Our finding that high ED consumption is more common among adolescents with a lower SES, who spend more leisure time in front of a screen and who are less physically active, suggests that for some adolescents, ED consumption may be an additional factor leading to potentially adverse effects on somatic and mental health and development. Even making no inferences on causality, it can be argued that it probably would be beneficial for these adolescents to reduce their ED intake.

### Strengths and limitations

There are some strengths and limitations to our study that should be taken into account.

There is always the risk of recall and reporting bias with self-reported data. However, in order to attain a large number of participants in this kind of cross-sectional study, the use of questionnaires is necessary. Our calculations of daily ED consumption are based on self-reported average frequency and volume, making this measure a crude semi-quantitative estimate. Non-participation is a challenge in epidemiologic studies [34] and it should be noted that eligible schools, classes or students who did not participate in the survey may have influenced the findings presented here. The average response rate for upper secondary schools was 51.2%, while it was 78.2% for lower secondary schools.

One asset of this study is that it covered a large geographical area, including both rural and urban areas. Furthermore, it employed a well-established survey [27, 35] in which all schools are invited to participate. The sample size is another major advantage of this study.

### Directions for future research

In the future, we suggest that researchers might consider using more continuous registration methods, such as daily registrations of ED consumption, physical activity and media use over a predefined period of time. We recommend that further experimental studies should be conducted to investigate the effects of ED consumption. There seems to be a lack of longitudinal studies in this research field, and systematic reviews are also warranted.

### Significance

The finding of widespread ED consumption among children and adolescents, as well as the characteristics of high-consumption groups, must be considered by national public health authorities in the perspective of a possible need for introducing regulations for the marketing and sale of these products. In addition, a focus on adolescents at risk of increased ED consumption should be established in primary and secondary health care, as well as in schools.

## Conclusion

In conclusion, this study surveyed the extent of ED consumption among adolescents in Norway, and assessed the impact of sociodemographic and lifestyle factors on ED consumption. More than half of the respondents reported that they were ED consumers. We found that daily and high ED consumption were independently associated with male gender, physical inactivity, high leisure screen time, low socioeconomic status and rural residency.

## Additional files

Additional file 1: Socioeconomic status. Description on how SES scores were assessed and computed. (DOCX 18 kb)

Additional file 2: **Table S5.** Geometric mean differences^a^ in daily ED consumption among ED consumers by gender and school level. (DOCX 21 kb)

Additional file 3: **Table S6.** Adjusted^a^ odds ratios (OR) for ED consumers by school level and by gender. (DOCX 16 kb)

Additional file 4: **Table S7.** Adjusted^a^ odds ratios (OR) for ED high consumers by school level and by gender. (DOCX 22 kb)

## Abbreviations

CI: Confidence interval
ED: Energy drink
OR: Odds ratio
SES: Socioeconomic status

## References

[1] Zucconi S, Volpato C, Adinolfi F, Gandini E, Gentile E, Loi A, et al. Gathering consumption data on specific consumer groups of energy drinks. Supporting Publications. 2013;10:EN-394:[190 pp.]. https://efsa.onlinelibrary.wiley.com/doi/abs/10.2903/sp.efsa.2013.EN-394. Accessed 13 Feb 2015.

[2] Seifert SM, Schaechter JL, Hershorin ER, Lipshultz SE (2011). Health effects of energy drinks on children, adolescents, and young adults. Pediatrics.

[3] Visram S, Cheetham M, Riby DM, Crossley SJ, Lake AA. Consumption of energy drinks by children and young people: a rapid review examining evidence of physical effects and consumer attitudes. BMJ Open 2016;6:(no pagination)(e010380). 10.1136/bmjopen-2015-010380 PMC5073652 27855083

[4] Harris JL, Munsell CR (2015). Energy drinks and adolescents: what's the harm?. Nutr Rev.

[5] Wolk BJ, Ganetsky M, Babu KM (2012). Toxicity of energy drinks. Curr Opin Pediatr.

[6] Kozik TM, Shah S, Bhattacharyya M, Franklin TT, Connolly TF, Chien W (2016). Cardiovascular responses to energy drinks in a healthy population: the C-energy study. Am J Emerg Med.

[7] Grasser EK, Yepuri G, Dulloo AG, Montani JP (2014). Cardio- and cerebrovascular responses to the energy drink red bull in young adults: a randomized cross-over study. Eur J Nutr.

[8] Svatikova A, Covassin N, Somers KR, Somers KV, Soucek F, Kara T (2015). A randomized trial of cardiovascular responses to energy drink consumption in healthy adults. JAMA.

[9] Christensen LM, Iversen JD, Biltoft-Jensen A, Petersen MA, Søndergaard AB, Matthiessen J. Consumption of energy drinks among 10–35-yr-old Danes (in Danish with an English summary). National Food Institute, Technical University of Denmark; 2014. http://www.food.dtu.dk/english/-/media/Institutter/Foedevareinstituttet/Publikationer/Pub-2014/Rapport-om-energidrikke-i-Danmark.ashx?la=da

[10] Kristjansson AL, Sigfusdottir ID, Mann MJ, James JE (2014). Caffeinated sugar-sweetened beverages and common physical complaints in Icelandic children aged 10-12 years. Prev Med.

[11] Koivusilta L, Kuoppamaki H, Rimpela A (2016). Energy drink consumption, health complaints and late bedtime among young adolescents. Int J Public Health.

[12] Dawodu A, Cleaver K (2017). Behavioural correlates of energy drink consumption among adolescents: a review of the literature. J Child Health Care.

[13] Troxel WM, Tucker JS, Ewing B, Miles JNV, D’Amico EJ (2018). Sleepy teens and energy drink use: results from an ethnically diverse sample of youth. Behav Sleep Med.

[14] Holubcikova J, Kolarcik P, Madarasova Geckova A, Reijneveld S, van Dijk J (2017). Regular energy drink consumption is associated with the risk of health and behavioural problems in adolescents. Eur J Pediatr.

[15] Kim SY, Sim S, Choi HG. High stress, lack of sleep, low school performance, and suicide attempts are associated with high energy drink intake in adolescents. PLoS One. 2017;12:(no pagination)(e0187759). 10.1371/journal.pone.0187759 PMC5685612 29135989

[16] Van Batenburg-Eddes T, Lee NC, Weeda WD, Krabbendam L, Huizinga M (2014). The potential adverse effect of energy drinks on executive functions in early adolescence. Front Psychol.

[17] Hardy LL, Bell J, Bauman A, Mihrshahi S (2018). Association between adolescents' consumption of total and different types of sugar-sweetened beverages with oral health impacts and weight status. Aust N Z J Public Health.

[18] Utter J, Denny S, Teevale T, Sheridan J (2018). Energy drink consumption among New Zealand adolescents: associations with mental health, health risk behaviours and body size. J Paediatr Child Health.

[19] Opinion of the French Agency for Food, Environmental and Occupational Health and Safety on the assessment of risks concerning the consumption of so-called “energy drinks”. 2013:[108 pp.]. https://www.anses.fr/sites/default/files/documents/NUT2012sa0212EN.pdf. Accessed 4 Feb 2016.

[20] Hammond D, Reid JL, Zukowski S (2018). Adverse effects of caffeinated energy drinks among youth and young adults in Canada: a web-based survey. CMAJ Open.

[21] Temple JL (2009). Caffeine use in children: what we know, what we have left to learn, and why we should worry. Neurosci Biobehav Rev.

[22] Reid JL, Hammond D, McCrory C, Dubin JA, Leatherdale ST (2015). Use of caffeinated energy drinks among secondary school students in Ontario: prevalence and correlates of using energy drinks and mixing with alcohol. Can J Public Health.

[23] Park S, Blanck HM, Sherry B, Brener N, O'Toole T (2012). Factors associated with sugar-sweetened beverage intake among United States high school students. J Nutr.

[24] Terry-McElrath YM, O'Malley PM, Johnston LD (2014). Energy drinks, soft drinks, and substance use among United States secondary school students. J Addict Med.

[25] Al-Hazzaa HM, Al-Sobayel HI, Abahussain NA, Qahwaji DM, Alahmadi MA, Musaiger AO (2014). Association of dietary habits with levels of physical activity and screen time among adolescents living in Saudi Arabia. J Hum Nutr Diet.

[26] Upper secondary education [Internet]. Statistisk sentralbyrå. 2018. https://www.ssb.no/en/vgu/. Accessed 7 May 2018.

[27] Ungdata [Internet]. 2016. http://www.ungdata.no/English. Accessed 11 June 2017.

[28] Bakken A, Frøyland LR, Sletten MA (2016). Sosiale forskjeller i unges liv. Hva sier Ungdata-undersøkelsene? [Social differences in young people’s lives. What can UNGDATA tell us?].

[29] Currie C, Molcho M, Boyce W, Holstein B, Torsheim T, Richter M (2008). Researching health inequalities in adolescents: the development of the health behaviour in school-aged children (HBSC) family affluence scale. Soc Sci Med.

[30] Faris MA, Epuru S, Al-Shimmari S, Al-Shimmari E (2015). Alarming high levels of energy drinks consumption among school children in hail, northern of Saudi Arabia. Int J Child Health Nutr.

[31] Larson N, DeWolfe J, Story M, Neumark-Sztainer D (2014). Adolescent consumption of sports and energy drinks: linkages to higher physical activity, unhealthy beverage patterns, cigarette smoking, and screen media use. J Nutr Educ Behav.

[32] Hale L, Guan S (2015). Screen time and sleep among school-aged children and adolescents: a systematic literature review. Sleep Med Rev.

[33] Hoare E, Milton K, Foster C, Allender S (2016). The association between sedentary behaviour and mental health among adolescents: a systematic review. Int J Behav Nutr Phys Act.

[34] Galea S, Tracy M (2007). Participation rates in epidemiologic studies. Ann Epidemiol.

[35] Ungdata 2010–2013. Metode og Dokumentasjon [Ungdata 2010–2013. Methods and Documentation], Oslo. http://www.ungdata.no/Forskning/Metode-og-dokumentasjon/Ungdata-2010-2013-Metode-og-dokumentasjon. Accessed 12 June 2017.

