# Supplementary material for: Consumption of energy drinks among adolescents in Norway: a cross-sectional study
Source: BMC Public Health. 2018 Dec 19;18:1391. doi: 10.1186/s12889-018-6236-5 (PMC6299924; doi:10.1186/s12889-018-6236-5)
Supplement: Supplementary file 1 — Socioeconomic status. Description on how SES scores were assessed and computed. (DOCX 18 kb) [file 12889_2018_6236_MOESM1_ESM.docx]

**Additional file 1**

**Socioeconomic status**

Socioeconomic status was attributed to a five-point scale, based on a compound score from three dimensions (27, 28). The first dimension concerned parental education. The question “Did your mother/father study at a university or at a university college?” was used (separate responses for mother and father: 3 points for “yes” on both, 1.5 points for one of them and 0 points for “no” on both, missing was attributed 0 points). The second dimension concerned “cultural capital” at home, measured by asking “How many books do you think there are in your home?” (options: “no books” = 0 points , “fewer than 20 books” = 0.6 points, “20-100 books” = 1.2 points, “100-500 books” = 1.8 points, “500-1000 books” = 2.4 points, “more than 1000 books” = 3 points). The third dimension concerned family affluence using the Family Affluence Scale (27), consisting of four questions on material resources in the family: “Does your family have a car?” (options: “no” = 0 points, “yes, one” = 2 points, “yes, two or more” = 3 points), “Do you have your own bedroom?” (options: “yes” = 3 points, “no” = 0 points), “How many times have you travelled somewhere on holiday with your family over the past year?” (options: “none” = 0 points, “once” = 1 point, “twice” = 2 points, “more than twice” = 3 points), “How many computers does your family have?” (options: “none” = 0 points, “one” = 1 point, “two” = 2 points, “more than two” = 3 points). First, an average of the four questions concerning Family Affluence Scale was calculated. Next, an average of all three socioeconomic dimensions was calculated with a subsequent distribution into five equally sized groups, where group 5 represents highest SES while group 1 represents lowest SES.
