# Supplementary material for: Consumption of energy drinks among adolescents in Norway: a cross-sectional study
Source: BMC Public Health. 2018 Dec 19;18:1391. doi: 10.1186/s12889-018-6236-5 (PMC6299924; doi:10.1186/s12889-018-6236-5)
Supplement: Supplementary file 2 — Table S5. Geometric mean differencesa in daily ED consumption among ED consumers by gender and school level. (DOCX 21 kb) [file 12889_2018_6236_MOESM2_ESM.docx]

| **Additional Table 5**  **Geometric mean differences^a^ in daily ED consumption among ED consumers by gender and school level** | | | | | | | | | | | |  |
| --- | --- | --- | --- | --- | --- | --- | --- | --- | --- | --- | --- | --- |
|  | |  | **Lower secondary school^b^** | |  | | **Upper secondary school^c^** | | | |  |  |
|  |  | | **Boys**  n = 5,654 | **Girls**  n = 3,615 |  | | | **Boys**  n = 3,384 | | **Girls**  n = 2,473 |  | |
| **Variable** | **GM (%) [95% CI]** | | | **GM (%) [95% CI]** | | **GM (%) [95% CI]** | | | **GM (%) [95% CI]** | | |  |
| Residency^d^ |  | | |  | |  | | |  | | |  |
| Urban |  | | |  | |  | | |  | | |  |
| Rural | 17.5 [10.2-25.3] | | | 10.6 [3.8-17.9] | | 10.4 [1.5-20.0] | | | 7.7 [-0.2-16.2] | | |  |
|  |  | | |  | |  | | |  | | |  |
| Socioeconomic status |  | | |  | |  | | |  | | |  |
| Group 5 Highest |  | | |  | |  | | |  | | |  |
| Group 4 | -5.9 [-15.2-4.3] | | | -7.1 [-16.3-3.1] | | 12.6 [-1.3-28.3] | | | 8.7 [-4.7-24.1] | | |  |
| Group 3 | 5.4 [-5.2-17.3] | | | 1.8 [-8.1-12.7] | | 1.3 [-11.3-15.7] | | | 28.2 [12.8-45.8] | | |  |
| Group 2 | 13.4 [2.0-26.0] | | | 4.6 [-5.4-15.6] | | 11.2 [-2.7-27.1] | | | 28.1 [13.2-45.0] | | |  |
| Group 1 Lowest | 8.4 [-2.4-25.3] | | | 29.3 [16.7-43.3] | | 10.0 [-4.0-26.0] | | | 35.2 [19.4-53.2] | | |  |
|  |  | | |  | |  | | |  | | |  |
| Frequency of physical activity^e^ |  | | |  | |  | | |  | | |  |
| Often |  | | |  | |  | | |  | | |  |
| Seldom | 16.3 [5.1-28.5] | | | 18.5 [8.4-29.5] | | 16.4 [2.7-31.8] | | | 0.3 [-8.9-10.3] | | |  |
| Never | 51.3 [18.7-93.0] | | | 117.0 [71.6-174.4] | | 62.3 [21.5-117.0] | | | 45.9 [12.5-89.1] | | |  |
|  |  | | |  | |  | | |  | | |  |
| Leisure screen time |  | | |  | |  | | |  | | |  |
| Less than two hours |  | | |  | |  | | |  | | |  |
| Two-three hours | 19.1 [7.6-31.8] | | | 16.0 [4.8-28.4] | | 22.6 [6.1-41.6] | | | 4.4 [-8.2-18.6] | | |  |
| Three-four hours | 40.6 [27.5-55.0] | | | 19.6 [8.5-31.8] | | 34.2 [16.8-54.2] | | | 12.0 [-1.1-26.8] | | |  |
| Four-six hours | 63.7 [47.5-81.7] | | | 36.1 [22.8-50.9] | | 69.9 [47.7-95.6] | | | 29.1 [13.4-47.1] | | |  |
| More than six hours | 155.8 [128.9-185.8] | | | 99.6 [78.3-123.5] | | 112.6 [83.8-145.8] | | | 49.3 [29.6-72.0] | | |  |

*Note:*  GM = Geometric mean difference; CI = confidence interval

^a^ Adjusted for the other variables in the table.

^b^ Lower secondary school includes grades 8-10 and ages 12-15 years.

^c^ Upper secondary school includes grades 11-13 and ages 15-19 years.

^d^ Urban residency: municipalities with > 20,000 residents, rural residency: municipalities with < 20 000 residents.

^e^ Often: once a week or more, seldom: once to twice a month or less.
