# Supplementary material for: Consumption of energy drinks among adolescents in Norway: a cross-sectional study
Source: BMC Public Health. 2018 Dec 19;18:1391. doi: 10.1186/s12889-018-6236-5 (PMC6299924; doi:10.1186/s12889-018-6236-5)
Supplement: Supplementary file 3 — Table S6. Adjusteda odds ratios (OR) for ED consumers by school level and by gender. (DOCX 16 kb) [file 12889_2018_6236_MOESM3_ESM.docx]

| **Additional Table 6**  **Adjusted^a^ odds ratios (OR) for ED consumers by school level and by gender** | | | | | | | | | | |  |
| --- | --- | --- | --- | --- | --- | --- | --- | --- | --- | --- | --- |
|  | | **Lower secondary school^b^** | |  | | **Upper secondary school^c^** | | | | |  |
|  |  | **Boys**  n = 9,030 | **Girls**  n = 9,343 |  | | | **Boys**  n = 5,221 | | **Girls**  n = 5,950 |  | |
| **Variable** | **OR [95% CI]** | | **OR [95% CI]** | | **OR [95% CI]** | | | **OR [95% CI]** | | |  |
| Residency^d^ |  | |  | |  | | |  | | |  |
| Urban | 1 | | 1 | | 1 | | | 1 | | |  |
| Rural | 1.19 [1.09-1.30] | | 1.22 [1.12-1.33] | | 1.21 [1.08-1.36] | | | 1.12[1.01-1.25] | | |  |
|  |  | |  | |  | | |  | | |  |
| Socioeconomic status |  | |  | |  | | |  | | |  |
| Group 5 Highest | 1 | | 1 | | 1 | | | 1 | | |  |
| Group 4 | 1.32 [1.15-1.52] | | 1.16 [1.01-1.32] | | 1.12 [0.94-1.33] | | | 1.17 [0.99-1.38] | | |  |
| Group 3 | 1.26 [1.10-1.46] | | 1.28 [1.11-1.46] | | 1.23 [1.03-1.48] | | | 1.42 [1.20-1.69] | | |  |
| Group 2 | 1.28 [1.11-1.47] | | 1.44 [1.26-1.65] | | 1.35 [1.12-1.62] | | | 1.79 [1.52-2.12] | | |  |
| Group 1 Lowest | 1.40 [1.21-1.61] | | 1.53 [1.33-1.76] | | 1.35 [1.12-1.63] | | | 1.98 [1.67-2.35] | | |  |
|  |  | |  | |  | | |  | | |  |
| Frequency of physical activity^e^ |  | |  | |  | | |  | | |  |
| Often | 1 | | 1 | | 1 | | | 1 | | |  |
| Seldom | 0.86 [0.75-0.99] | | 1.01 [0.89-1.15] | | 0.90 [0.76-1.08] | | | 1.00 [0.87-1.14] | | |  |
| Never | 0.82 [0.59-1.15] | | 0.95 [0.68-1.32] | | 0.84 [0.56-1.25] | | | 1.22 [0.82-1.82] | | |  |
|  |  | |  | |  | | |  | | |  |
| Leisure screen time |  | |  | |  | | |  | | |  |
| Less than two hours | 1 | | 1 | | 1 | | | 1 | | |  |
| Two-three hours | 1.63 [1.44-1.85] | | 1.56 [1.38-1.77] | | 1.44 [1.20-1.72] | | | 1.36 [1.15-1.60] | | |  |
| Three-four hours | 2.02 [1.79-2.29] | | 2.09 [1.84-2.36] | | 1.60 [1.34-1.90] | | | 1.66 [1.41-1.95] | | |  |
| Four-six hours | 2.32 [2.02-2.67] | | 2.92 [2.54-3.36] | | 1.85 [1.54-2.22] | | | 1.86 [1.56-2.21] | | |  |
| More than six hours | 2.94 [2.51-3.44] | | 4.01 [3.40-4.73] | | 2.42 [1.98-2.96] | | | 2.53 [2.07-3.09] | | |  |

*Note:* OR = Odds ratio; CI = Confidence interval

^a^ Adjusted for the other variables in the table.

^b^ Lower secondary school includes grades 8-10 and ages 12-15 years

^c^ Upper secondary school includes grades 11-13 and ages 15-19 years

^d^ Urban residency: municipalities with > 20,000 residents, rural residency: municipalities with < 20,000 residents.

^e^ Often: once a week or more, seldom: once to twice a month or less
