# Supplementary material for: Consumption of energy drinks among adolescents in Norway: a cross-sectional study
Source: BMC Public Health. 2018 Dec 19;18:1391. doi: 10.1186/s12889-018-6236-5 (PMC6299924; doi:10.1186/s12889-018-6236-5)
Supplement: Supplementary file 4 — Table S7. Adjusteda odds ratios (OR) for ED high consumers by school level and by gender. (DOCX 22 kb) [file 12889_2018_6236_MOESM4_ESM.docx]

| **Additional Table 7**  **Adjusted^a^ odds ratios (OR) for ED high consumers by school level and by gender** | | | | | | | | | | |  |
| --- | --- | --- | --- | --- | --- | --- | --- | --- | --- | --- | --- |
|  | | **Lower secondary school^b^** | |  | | **Upper secondary school^c^** | | | | |  |
|  |  | **Boys**  n = 9,030 | **Girls**  n = 9,343 |  | | | **Boys**  n = 5,221 | | **Girls**  n = 5,950 |  | |
| **Variable** | **OR [95% CI]** | | **OR [95% CI]** | | **OR [95% CI]** | | | **OR [95% CI]** | | |  |
| Residency^d^ |  | |  | |  | | |  | | |  |
| Urban | 1 | | 1 | | 1 | | | 1 | | |  |
| Rural | 1.19 [0.98-1.45] | | 1.68 [1.14-2.46] | | 1.40 [1.11-1.77] | | | 1.23 [0.81-1.86] | | |  |
|  |  | |  | |  | | |  | | |  |
| Socioeconomic status |  | |  | |  | | |  | | |  |
| Group 5 Highest | 1 | | 1 | | 1 | | | 1 | | |  |
| Group 4 | 0.90 [0.64-1.26] | | 0.61 [0.29-1.32] | | 1.21 [0.83-1.75] | | | 2.26 [0.86-5.92] | | |  |
| Group 3 | 1.18 [0.85-1.64] | | 1.16 [0.62-2.18] | | 0.98 [0.66-1.46] | | | 2.91 [1.15-7.39] | | |  |
| Group 2 | 1.20 [0.87-1.65] | | 1.33 [0.72-2.44] | | 1.21 [0.83-1.77] | | | 3.96 [1.62-9.65] | | |  |
| Group 1 Lowest | 1.25 [0.91-1.72] | | 1.85 [1.03-3.31] | | 1.17 [0.80-1.72] | | | 3.84 [1.56-9.43] | | |  |
|  |  | |  | |  | | |  | | |  |
| Frequency of physical activity^e^ |  | |  | |  | | |  | | |  |
| Often | 1 | | 1 | | 1 | | | 1 | | |  |
| Seldom | 1.44 [1.11-1.85] | | 1.47 [0.95-2.29] | | 1.50 [1.12-2.02] | | | 1.17 [0.73-1.89] | | |  |
| Never | 2.09 [1.28-3.43] | | 6.04 [3.30-11.06] | | 2.01 [1.12-3.58] | | | 1.31 [0.40-4.32] | | |  |
|  |  | |  | |  | | |  | | |  |
| Leisure screen time |  | |  | |  | | |  | | |  |
| Less than two hours | 1 | | 1 | | 1 | | | 1 | | |  |
| Two-three hours | 1.09 [0.76-1.57] | | 1.28 [0.57-2.88] | | 1.40 [0.88-2.22] | | | 1.15 [0.47-2.83] | | |  |
| Three-four hours | 1.54 [1.10-2.15] | | 2.41 [1.19-4.89] | | 1.28 [0.81-2.02] | | | 1.97 [0.87-4.45] | | |  |
| Four-six hours | 1.67 [1.18-2.37] | | 3.27 [1.59-6.76] | | 2.23 [1.45-3.40] | | | 2.94 [1.32-6.56] | | |  |
| More than six hours | 5.00 [3.66-6.84] | | 10.82[5.58-20.99] | | 3.32 [2.19-5.05] | | | 4.91 [2.19-11.0] | | |  |

*Note:* OR = Odds ratio; CI = Confidence interval. High consumer defined as drinking ED four times or more weekly.

^a^ Adjusted for the other variables in the table.

^b^ Lower secondary school includes grades 8-10 and ages 12-15 years.

^c^ Upper secondary school includes grades 11-13 and ages 15-19 years.

^d^ Urban residency: municipalities with > 20,000 residents, rural residency: municipalities with < 20,000 residents.

^e^ Often: once a week or more, seldom: once to twice a month or less.
